# A Network-Theory-Based Comparative Study of Melt-Conveying Models in Single-Screw Extrusion: A. Isothermal Flow

**DOI:** 10.3390/polym10080929

**Published:** 2018-08-19

**Authors:** Christian Marschik, Wolfgang Roland, Jürgen Miethlinger

**Affiliations:** Institute of Polymer Extrusion and Compounding, Johannes Kepler University Linz, 4040 Linz, Austria; wolfgang.roland@jku.at (W.R.); juergen.miethlinger@jku.at (J.M.)

**Keywords:** modeling and simulation, polymer processing, extrusion

## Abstract

In many extrusion processes, the metering section is the rate-controlling part of the screw. In this functional zone, the polymer melt is pressurized and readied to be pumped through the die. We have recently proposed a set of heuristic models for predicting the flow behavior of power-law fluids in two- and three-dimensional metering channels. These novel theories remove the need for numerical simulations and can be implemented easily in practice. Here we present a comparative study designed to validate these new methods against experimental data. Extensive experiments were performed on a well-instrumented laboratory single-screw extruder, using various materials, screw designs, and processing conditions. A network-theory-based simulation routine was written in MATLAB to replicate the flow in the metering zones in silico. The predictions of the three-dimensional heuristic melt-conveying model for the axial pressure profile along the screw are in excellent agreement with the experimental extrusion data. To demonstrate the usefulness of the novel melt-flow theories, we additionally compared the models to a modified Newtonian pumping model known from the literature.

## 1. Introduction

The extruder is the most important processing machine in the polymer industry. Every year, this type of machinery converts more than 114 million tons of raw polymeric materials [1]. In addition to producing semi-finished products such as pipes, films, profiles, cables, fibers, coatings, and sheets, the processing machine is frequently employed in compounding and recycling operations. Furthermore, it is used as a feeding unit in various polymer-shaping processes, such as injection molding, blow molding, and thermoforming. Consequently, a substantial proportion of polymers passes through an extrusion line at least once after manufacture. The dominance of the screw-barrel configuration in the polymer-processing field is due to its continuous development over the past few decades. This technical progress has gone hand in hand with extensive theoretical and experimental research. Thorough reviews of the process were given in [2,3,4,5,6].

The research reported here investigates the single-screw extrusion process, which can be divided into several functional steps. One of these processes involves melt-conveying and pressurization. To force the polymer melt through the die at the desired processing rate, the extruder must pump the material and build up sufficient pressure. Depending on screw and barrel design, pressure development may take place in all functional processing zones at different levels. The pressure profile along the screw channel can therefore exhibit various characteristics. In many industrial applications (e.g., in smooth-bore extrusion or in melt-fed extrusion), the metering section shows a pressure-generating, conveying behavior. As a result, the flow in the screw channel is subject to a positive pressure gradient that reduces the net flow. In other processes (e.g., in grooved-feed extrusion), the solids-conveying section builds up a substantial level of pressure, and the downstream functional zones are overridden. The flow in the screw channel is governed by a negative pressure gradient that increases the net flow.

Numerous theoretical studies have investigated melt-conveying and pressurization in single-screw extruders. These analyses can be classified by their level of complexity into the following categories and their combinations: (i) Newtonian and non-Newtonian models; (ii) one-dimensional and multi-dimensional models; (iii) isothermal and non-isothermal models. The first model of screw viscosity pumps was published anonymously [7] and later extended by Rowell and Finlayson [8]. They analyzed the isothermal conveying characteristics of a Newtonian fluid between parallel boundaries in linear movement and provided the first insights into the flow mechanisms in a straight, rectangular screw channel. Similarly, Carley et al. [9] developed a simplified flow theory for screw extruders, placing special emphasis on tapered screw channels. To investigate distributive mixing, Mohr et al. [10,11] examined the transverse flow in a metering channel of infinite width.

These studies dealt with Newtonian fluids. Assuming the viscosity of the polymer melt to be constant, the theories proposed exact analytical solutions for the cross-channel and down-channel flows. Further, to describe the pumping capability of the melt-conveying zone, the flow rate was expressed as a linear superposition of a drag and a pressure flow.

Including the shear-thinning flow behavior of polymer melts in the analysis of melt-conveying and pressurization involves the use of numerical procedures. For non-Newtonian fluids, the governing flow equations become non-linear due to the dependence of viscosity on shear rate. Physically, this means that the drag and the pressure flow are coupled and cannot be analyzed independently. In multidimensional problems, complexity is further increased by the combined effect of shear in the down- and cross-channel directions. A well-known mathematical relationship that is commonly applied to describe the shear-thinning behavior of polymer melts is the power-law model according to Ostwald-deWaele [12], which relates the viscosity and the shear-rate via two independent material parameters: (i) The consistency and (ii) the power-law index of the polymer melt. Even for the most simplified mathematical problem—a temperature-independent flow of a power-law fluid between two parallel plates—no exact analytical solution has been found to date, and hence numerical methods are essential for accurate flow analyses in single-screw extruders [6]. Several studies have investigated the flow of power-law fluids in screw viscosity pumps. Rotem and Shinnar [13] presented numerical results for a one-dimensional flow under isothermal conditions. Griffith [14], Zamodits and Pearson [15], Booy [16], and Karwe and Jaluria [17] obtained numerical solutions for a two-dimensional flow in a screw channel of infinite width, taking the effect of the transverse flow on the pumping behavior into account. An alternative approach was proposed by Middleman [18]. He calculated the drag and pressure flows separately, and superimposed the results to point out the influence of the screw flights on the flow in rectangular ducts. Spalding et al. [19] investigated a three-dimensional flow in a helical screw channel by using the finite-element method.

To remove the need for time-consuming numerical procedures, a few studies have proposed analytical approximation methods for estimating the effect of the shear-thinning flow behavior on the pumping capability of the extruder, as summarized in Table 1. Booy [16] applied the classical Newtonian pumping model to derive effective viscosity values and approximate shear-thinning flow behavior. White and Potente [3] developed an independent discontinuous modeling approach to predicting the pumping capability of two-dimensional screw channels. A continuous approximation method for pressure-generating metering sections was proposed by Rauwendaal [6]. He applied correction factors to the drag- and pressure flows in the classical pumping model to include the non-Newtonian flow behavior.

Our research group has recently presented a heuristic method for modeling the flow of power-law fluids in metering channels. The novelty of this approach lies in the construction of heuristic models from a large number of numerical solutions to scaled flow equations and the use of symbolic regression based on genetic programming. Pachner et al. [20] published an isothermal melt-conveying model for two-dimensional metering channels. Roland and Miethlinger [22,23] provided approximate equations for calculating the viscous dissipation in one- and two-dimensional metering zones. In [21,24,25], we introduced a three-dimensional isothermal melt-conveying model for single-screw extruders, considering both pressure-generating and pressure-consuming screw zones. In contrast to previous two-dimensional modeling approaches which investigated screw channels of infinite width, the novel three-dimensional melt-flow theory additionally includes the effect of the flight flanks on the pumping behavior.

The work described here was designed to test our two- and three-dimensional heuristic pumping models [20,21] against experimental data. In the first part of the study, experiments were carried out to obtain extrusion data for various screw designs, materials, and processing conditions. In the second part, a network-theory-based flow-simulation routine using our novel melt-flow theories was written to replicate the conveying characteristics of the metering zones in silico. For convenience, the design of the heuristic melt-conveying models is revisited in the modeling section.

## 2. Experimental

### 2.1. Materials

Five polyolefins with different rheological behaviors were compared in this study. Table 2 summarizes the melt-flow rates (MFR) (ranging from 0.25 to 15.0 g/10 min) and the main application fields of the materials. To determine the viscosity functions of the polymer melts, rheological measurements at two temperatures were carried out using an Anton-Paar MCR 302 plate-plate rheometer (Anton Paar, Graz, Austria).

**Figure 1:**
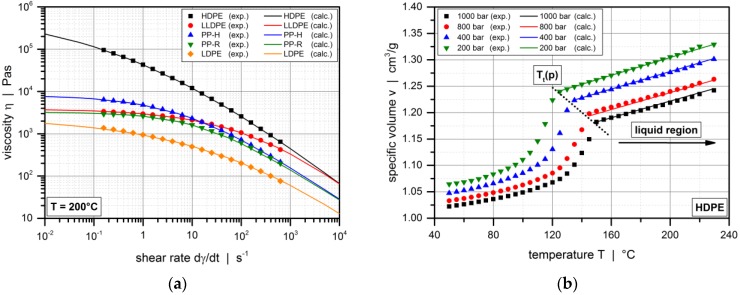
Comparison between experimental and calculated values: (**a**) Viscosity data of the polymer melts at temperature of 200 °C; (**b**) pressure-volume-temperature behavior of HDPE of pipe grade.

The experimental viscosity data were described mathematically by a temperature-dependent Carreau-Yasuda model [26,27]:

(1) $$\eta_{\mathrm{cy}}\left(\dot{\gamma },Τ\right)=a_{t}\eta_{\infty }+a_{t}\left(\eta_{0}-\eta_{\infty }\right){\left(1+{\left(a_{t}\lambda \dot{\gamma }\right)}^{a}\right)}^{\frac{n_{\mathrm{cy}}-1}{a}}$$

where *η*_0_ and *η*_∞_ are the viscosities at zero shear rate and at infinite shear rate, respectively, *λ* is the characteristic relaxation time, and *n*_cy_ the Carreau-Yasuda power-law index. This widely used viscosity function approximates the viscosity behaviors in the terminal and shear-thinning regimes, thus allowing accurate prediction of the viscosity characteristics over the whole range of shear rates. The temperature-shift factor was calculated by:

(2) $$a_{t}=\exp(-\alpha (T-T_{0}))$$

where *α* is the temperature coefficient of the viscosity and *T*_0_ the reference temperature. Table 3 lists the Carreau-Yasuda parameter values inferred from the experimental viscosity data. A comparison between experimental and calculated viscosity functions at a temperature of 200 °C is shown in Figure 1a.

Furthermore, by means of a Göttfert Rheograph 25 high-pressure capillary rheometer (GÖTTFERT, Buchen, Germany), the specific volumes of the materials tested were measured as functions of temperature and pressure. The pressure–volume–temperature (pvT) behavior was approximated mathematically by a Tait equation in the form of [28,29]:

(3) $$v\left(T,p\right)=v_{0}\left(T\right)\left(1-0.0894\ln\left(1+\frac{p}{B\left(T\right)}\right)\right)$$

where *v*_0_(*T*) is the specific volume at zero gauge pressure and *B*(*T*) accounts for the pressure sensitivity of the material. Focusing on the thermodynamic properties in the upper temperature region above the pressure-dependent transition temperature (*T* > *T*_t_(*p*)), we calculated the model parameters by:

(4) $$v_{0}\left(T\right)=b_{1m}+b_{2m}\left(T-b_{5m}\right)$$

(5) $$B\left(T\right)=b_{3m}\exp\left(-b_{4m}\left(T-b_{5m}\right)\right)$$

(6) $$T_{t}\left(p\right)=b_{5m}+b_{6m}p$$

where *b*_1m_–*b*_6m_ are coefficients. Table 3 shows the Tait parameter values fitted to the experimental pvT data. A comparison between experimental and calculated results in the liquid region is shown in Figure 1b for HDPE (high-density polyethylene) of pipe grade.

### 2.2. Equipment and Procedure

**Figure 2:**
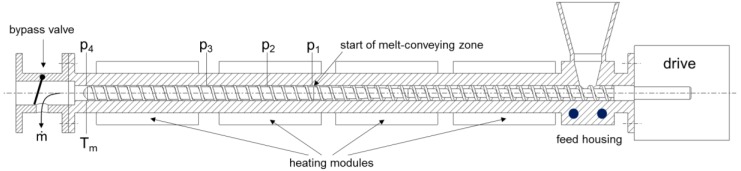
Schematic diagram of the plasticating single-screw extruder.

Experimental studies were performed on a Thermo Scientific HAAKE Rheomex 19/33 OS plasticating single-screw extruder (Fisher Scientific, Vienna, Austria). A schematic of the test setup is shown in Figure 2.

The laboratory extruder was equipped with a barrel of diameter *D*_b_ = 19.1 mm and axial length *L* = 619.35 mm (32.4·*D*_b_) measured from the front edge of the hopper. Thermal energy was supplied by electrical heaters that were clamped onto the barrel surface and grouped into four independent heating zones. A water-cooled feed housing was used to control the extruder temperature in the flow-in zone, while the remaining barrel sections were provided with forced-air cooling. Table 4 shows the barrel temperatures for the materials tested. A uniform temperature profile was chosen in each case to accelerate melting at the beginning of the screw and to keep the process as isothermal as possible. At the extruder head, a bypass valve was installed to discharge the polymer melt.

**Figure 3:**
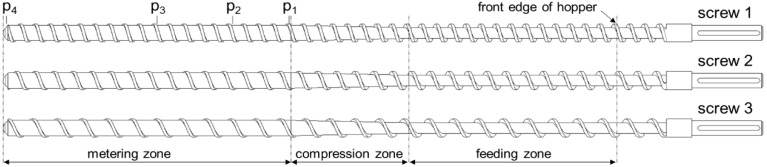
Schematic diagram of the extruder screws.

We employed three single-flighted standard extruder screws, each of which consisted of a feeding section (*L*_f_ = 209.85 mm), a compression section (*L*_c_ = 119.0 mm), and a melt-conveying section (*L*_m_ = 290.5 mm). The three screw designs differed only in screw pitch (Figure 3), which was varied (i) to allow different levels of transverse flow in the melt-conveying zone and (ii) to determine the effect of the screw flights on the flow rate. The former is governed by the pitch-to-diameter ratio of the screw, whereas the latter is driven by the aspect ratio of the screw channel. A detailed discussion of the influence of these dimensionless parameters on the pumping behavior of single-screw extruders can be found elsewhere [21]. Table 5 lists the dimensions of the metering channels under consideration.

To examine the pumping behavior of the screws, we located four pressure transducers with a measuring range of 0 to 500 bar along the barrel at the following axial positions: *L*_p1_ = 330.55 mm (17.3·*D*_b_), *L*_p2_ = 387.55 mm (20.3·*D*_b_), *L*_p3_ = 464.05 mm (24.3·*D*_b_), and *L*_p4_ = 616.05 mm (32.3·*D*_b_). Note that the position of the first sensor is very close to the geometric beginning of the melt-conveying zone, while the last sensor is positioned at the screw tip (Figure 3). The discharge temperature of the polymer melt was measured by means of a melt-temperature sensor at the end of the extruder.

Experiments were performed by increasing the screw speed from 25 to 250 rpm. With the bypass valve fully open, the extruder was operated at close to open discharge. For each operating point, the mass flow rate, the axial pressure profile, and the melt temperature were measured.

**Figure 4:**
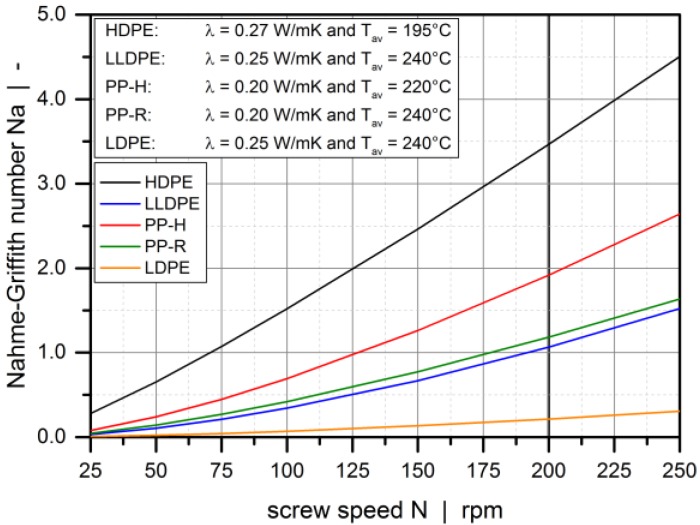
The Nahme–Griffith number as a function of screw speed for all materials.

To estimate the non-isothermal behavior of the flow in the metering zone, the Nahme–Griffith number was evaluated for each operating point. This characteristic ratio provides a dimensionless measure of the viscous heating relative to conduction in the radial direction [29]:

(7) $$Na=\frac{\alpha \eta_{\mathrm{av}}\;{\dot{\gamma }}_{\mathrm{av}}^{2}\;h^{2}}{\lambda }\;\mathrm{with}\;{\dot{\gamma }}_{\mathrm{av}}=\frac{D_{s}\pi N}{h}$$

where *η*_av_ is the average viscosity according to the Carreau-Yasuda model in Equation (1). In general, the range of the Nahme–Griffith number is 0 < *Na* < 200 [30]. Viscous heating and conduction is in balance if *Na* = 1.0, while heat generation is dominant if *Na* >> 1.0. Figure 4 shows the Nahme–Griffith number as a function of screw speed for all materials. With the channel height being constant for all screw designs, as indicated in Table 5, the results shown below are valid for all extruder screws.

Up to a screw speed of 200 rpm, the process can be considered isothermal for a majority of operating points. With *Na* < 1.0, viscous dissipation will not lead to temperature changes sufficient to affect viscosity. Non-isothermal behavior is observed in the case of HDPE. Due to its high molecular weight, the material shows pronounced viscous heating for large screw speeds. Note that the above results are highly dependent on the calculation of the average shear rate, which would be significantly reduced if the parameter was predicted component-wise. Based on the results in Figure 4, we omitted screw speeds above 200 rpm. Our main intention was to test the heuristic pumping models against a diversity of screw designs, materials, and operating conditions. Table A1 in the Appendix A shows the experimental setups used in the modeling section, including the measured processing data.

**Figure A1:**
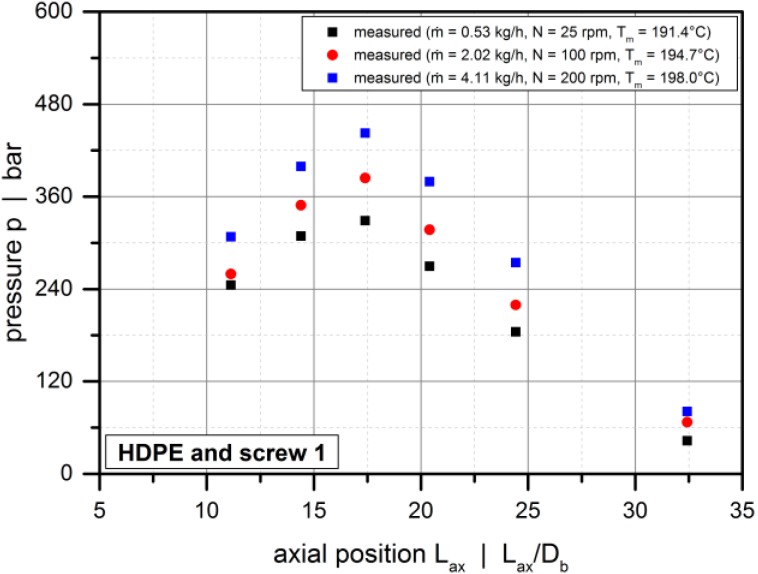
Experimental pressure profiles for HDPE and screw 1.

At the beginning of the experimental procedure, we performed pilot tests to investigate the degree of filling of the screw channel. Due to its small dimensions, the main objective was to examine if the channel is fully filled with pellets. To this end, additional pressure transducers were installed along the compression section of the screw. Figure A1 in the Appendix A illustrates axial pressure profiles for screw 1 and HDPE. The rapid increase in pressure along the early screw sections indicate the flood-feeding of the extruder.

**Figure A2:**
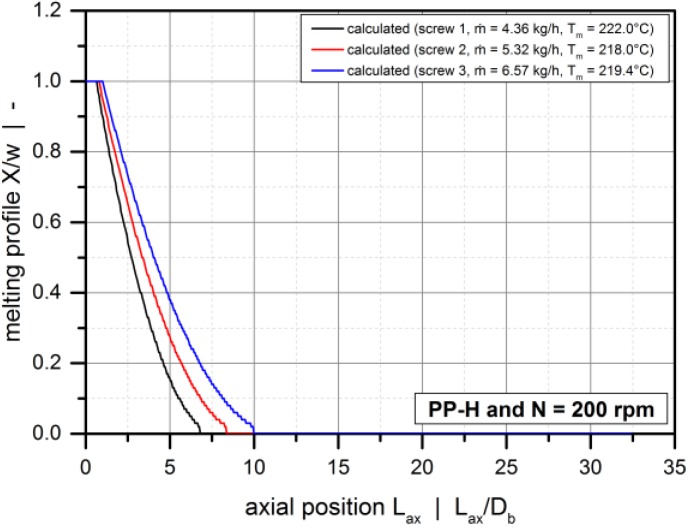
Calculated melting profiles for PP-H and *N* = 200 rpm.

Furthermore, to estimate the melting performance for the selected operating points, we carried out example calculations based on the well-known melting model proposed by Tadmor [4]. Given the melting profiles obtained from this study, we assumed that melting is almost or entirely completed at the beginning of the metering zone for most of the experimental setups, so that the solid content is negligibly small. Figure A2 in the Appendix A compares the melting characteristics for PP-H and a screw speed of 200 rpm for all three screw designs. Similar curves were obtained for other material and processing conditions. At this point, we would like to draw attention to the small channel height in the feeding zone of 3.9 mm (Table 5), compared to the average granule diameter of roughly 4.5 mm, causing the pellets to be compressed and heated up at the very beginning. Besides, melting is additionally accelerated by the barrel temperature profile, showing relatively high temperatures in the early screw sections (Table 4).

## 3. Modeling and Simulation

### 3.1. Heuristic Melt-Conveying Models

This section covers the fundamentals of the two- and three-dimensional heuristic melt-conveying models validated in this study. Detailed information can be found in [20,21]. In the following analysis, these two approaches are referred to as model 2D and model 3D. Table 6 compares their main characteristics.

#### 3.1.1. Problem Definition

**Figure 5:**
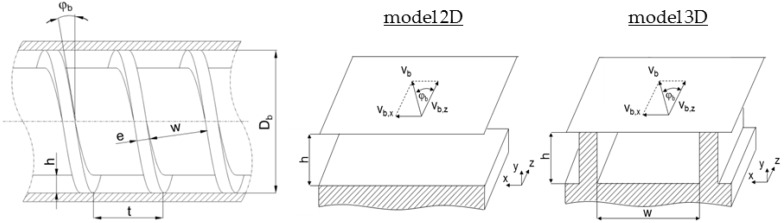
Representation of the screw channels in the two- and three-dimensional heuristic pumping models.

The heuristic pumping models under consideration are based on the flat-plate assumption: The helical screw channel is unwound from the screw and located on an infinite flat plate that represents the barrel surface, as illustrated in Figure 5. Avoiding the curvature of the screw, we employ a Cartesian coordinate system with *x*, *y*, and *z* denoting the cross-channel, up-channel, and down-channel directions, respectively. Note that for deep metering channels the screw curvature may have a considerable effect on the pumping behavior, as discussed in [31]. In addition, the kinematic conditions were reversed, assuming the screw channel to be fixed and the barrel surface to be moving at circumferential speed *v*_b_, which is decomposed into components in the cross- and down-channel directions, *v*_b,x_ and *v*_b,z_. The validity of this modeling approach has been thoroughly discussed in the literature [32]. In contrast to model 2D, which considers a screw channel of infinite width, model 3D includes the influence of flight flanks on both sides of the screw channel. Considering a fully developed flow, the velocity fields were defined as **v** = (*v*_x_(*y*), 0, *v*_z_(*y*))^T^ and **v** = (*v*_x_(*x*, *y*), *v*_y_(*x*, *y*), *v*_z_(*x*, *y*))^T^ in model 2D and model 3D, respectively.

The shear-thinning flow behavior was modeled as a power-law fluid:

(8) $$\eta_{\mathrm{pl}}\left(\dot{\gamma }\right)=K\cdot {\left|\dot{\gamma }\right|}^{n_{\mathrm{pl}}-1}$$

where *K* is the consistency and *n*_pl_ is the power-law index. Table 6 shows the simplified flow equations for both models. Time-dependent and thermal effects were omitted, and the governing flow equations were reduced to Stokes flow, as the flow of polymer melts is governed predominantly by internal friction rather than by inertial forces.

#### 3.1.2. Theory of Similarity and Numerical Solution

In the next step, the flow equations were rewritten in dimensionless form using the theory of similarity and dimensional analysis to identify the characteristic dimensionless groups of each flow situation. In model 2D, three independent dimensionless input parameters were identified: (i) The pitch-to-diameter ratio of the screw *t*/*D*_b_, (ii) the power-law index of the polymer melt *n*_pl_, and (iii) the dimensionless pressure gradient in the down-channel direction *Π*_p,z_ defined as:

(9) $$\Pi_{p,z}=\frac{p_{z}^{\prime }\;h^{1+n}}{6Kv_{b,z}^{n}}$$

where *p*_z_^′^ is the pressure gradient in the down-channel direction. In model 3D, a fourth independent dimensionless input parameter was found: (iv) The aspect ratio of the screw channel *h*/*w*. In general, two systems with the same dimensionless values are similar in terms of their underlying physics. Applying the theory of similarity therefore allows us to recognize arbitrary flow situations that may operate under different sets of flow conditions, but are governed by the same physics. Conversely, varying the dimensionless values of the characteristic scales allows us to alter the physical conditions of the flow.

Two sets of roughly 10,000 and 88,000 physically independent design points were created for the two- and three-dimensional flow situations, respectively, whose volume flow rates were calculated numerically. The scope of variation has been described elsewhere [20,21]. Similarly, the numerical results were rewritten in the form of a dimensionless flow rate *Π*_v_:

(10) $$\Pi_{v}=\frac{2\dot{V}}{iwhv_{b,z}}$$

#### 3.1.3. Heuristic Analysis

To remove the need for numerical procedures, the numerical solutions of the parametric design studies were approximated analytically, using symbolic regression based on genetic programming. This data-based modeling technique searches the space of mathematical expressions to find a symbolic function that relates sets of input and output data. In contrast to other modern regression methods, the modeling approach requires neither model structure nor model parameters to be predefined. Rather, by employing evolutionary computation, the method infers the regression model that best fits the given data set in terms of simplicity and accuracy from the data itself [33].

For each flow situation, a symbolic regression model was derived by applying the calculated datasets to a heuristic optimization algorithm. The dimensionless volume flow rate (*Π*_v_) was expressed as a function of the corresponding sets of independent dimensionless input parameters:

(11) $$\mathrm{model}2D:\;\Pi_{v}=f\left(\frac{t}{D_{b}},\;n,\;\Pi_{p,z}\right)\;\mathrm{and}\;\mathrm{model}\;3D:\;\Pi_{v}=f\left(\frac{h}{w},\;\frac{t}{D_{b}},\;n,\;\Pi_{p,z}\right)$$

A global error analysis was performed, which showed that the analytical approximations accurately predict the numerical solutions. The heuristic melt-conveying models thus allow rapid prediction of the isothermal conveying behavior of power-law fluids in two- and three-dimensional metering channels without the need for numerical procedures.

**Figure 6:**
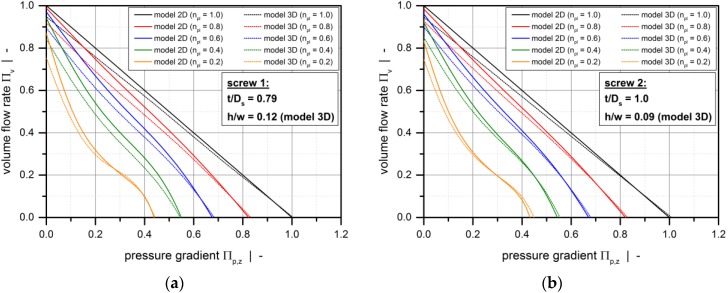

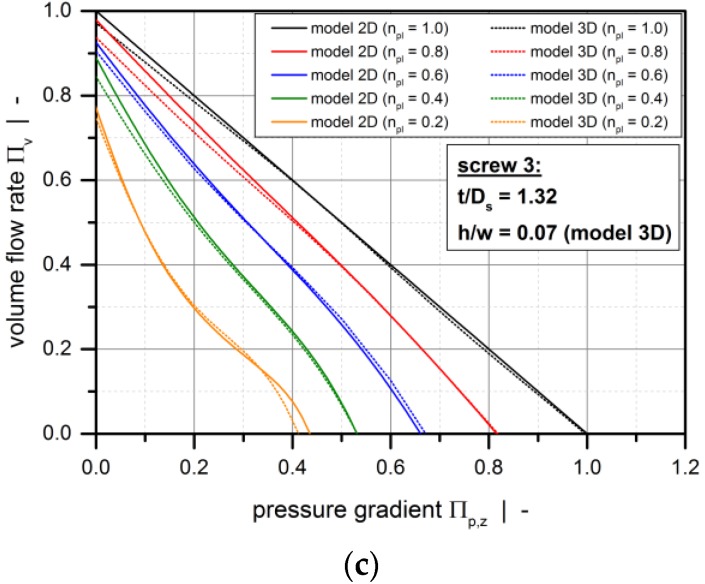
Comparison between model 2D and model 3D: Screw characteristic curves for the metering channels experimentally investigated in the first part (Table 5) and various power-law indices: Screw 1 (**a**), screw 2 (**b**), and screw 3 (**c**).

Figure 6 compares the heuristic pumping models, showing screw characteristic curves for the metering channels experimentally investigated in the first part and various power-law indices. To point out the influence of the screw flights on the pumping behavior, the aspect ratio in model 3D was set to *h*/*w* = 0.12 (a), *h*/*w* = 0.09 (b), *h*/*w* = 0.07 (c), as given in Table 5. Note that the screw sections compared below additionally exhibit different pitch-to-diameter ratios, which is a measure of the level of transverse flow in the melt-conveying zone.

Whereas the results for the shallow screw channel with *h*/*w* = 0.07 nearly coincide with the solutions of model 2D, a more significant difference is obvious if a boxier flow channel is defined. Given a pressure-generating melt-conveying section with *Π*_p,z_ > 0, the flow rate is reduced if the influence of the screw flights is taken into account.

In most standard extruder screws, the aspect ratio of the screw channel ranges from 0.05 to 0.15. In these designs, the restricting effect of the flights on flow is limited. For many multi-flighted high-performance screws (e.g., barrier screws), however, the ratio of channel depth to channel width exceeds 0.15, which requires the flow rate to be corrected. To include the effect of the screw flights on the flow in model 2D, we applied the following approximate shape factors derived from the classical Newtonian pumping model [2]:

(12) $$F_{d}=\frac{16w}{\pi^{3}h}\sum_{i=1,3,5}^{\infty }\frac{1}{i^{3}}\tanh\left(\frac{i\pi h}{2w}\right)\approx 0.1342{\left(\frac{h}{w}\right)}^{2}-0.6412\left(\frac{h}{w}\right)+1.0115$$

(13) $$F_{p}=1-\frac{192h}{\pi^{5}w}\sum_{i=1,3,5}^{\infty }\frac{1}{i^{5}}\tanh\left(\frac{i\pi w}{h}\right)\approx 0.1632{\left(\frac{h}{w}\right)}^{2}-0.7503\left(\frac{h}{w}\right)+1.0143$$

The implementation of the shape factors shown above in a network-theory-based flow-simulation routine is explained in the next section.

### 3.2. Network-Theory-Based Modeling

Recently, the heuristic pumping models have been tested successfully against additional numerical solutions not used in their design [25]. To further validate the methods against experimental data, a network-theory-based simulation routine was written in MATLAB. The objective was to replicate the flow in the metering zones experimentally analyzed in the first part of the study by predicting the axial pressure profile along the metering section for the experimental setups shown in Table A1.

Network theory originates from the field of electrical engineering [34], but has also proven useful in predicting the flow in extrusion dies [35] and in investigating the pumping behavior of barrier-screws [36]. The main idea is to model the flow in complex geometries by subdividing the system into geometrically simpler, interconnected elements for which analytical equations are available. The resulting two-dimensional equivalent circuit diagram is solved numerically by means of nodal analysis in which the currents are replaced with flow rates and the voltages with pressure differences. For non-Newtonian flows, an iterative procedure is additionally required to obtain converging solutions. Network theory provides a convenient modeling approach to consider local changes in geometry or material properties in the flow calculation.

Note that the heuristic melt-conveying models examined in this study consider an isothermal, incompressible, fully-developed flow of a power-law fluid in a screw channel of constant channel height, as indicated in Table 6. Thus, the application of network theory is required, for two reasons. First, our analysis is based on a pressure-dependent density. Since the pressure varies with the position in the screw channel, the melt density must be calculated incrementally. Second, our analysis includes leakage flow over the screw flights. Applying network theory allows us to capture the change in channel height at the flight clearances and consequently to describe the entire channel geometry accurately.

**Figure 7:**
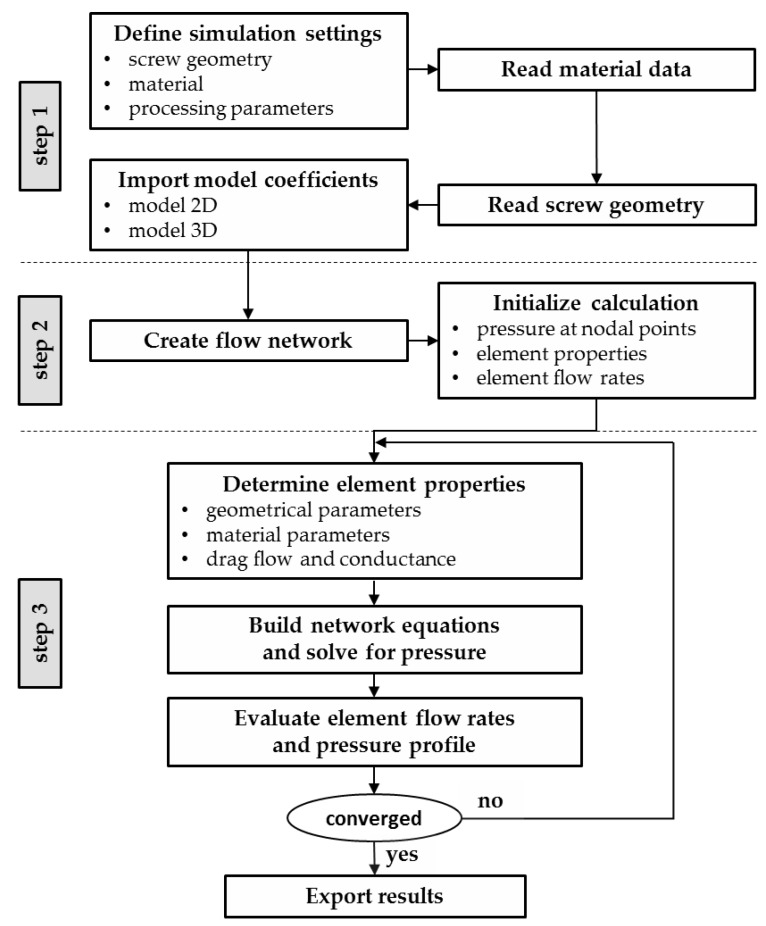
Flow chart of the screw-simulation routine.

Our simulation routine is based on the procedure illustrated in Figure 7. In the first step, basic simulation settings are defined. These include screw geometry (metering section), material of interest and input processing parameters, such as screw speed, mass flow rate, and melt temperature, which were evaluated experimentally. The last of these is used to shift the viscosity data to the desired temperature. For convenience, the geometric parameters of the metering sections (Table 5) and the flow properties of the materials (Table 3) are stored in an external library. To describe the shear-thinning nature of the polymer melts, the Carreau-Yasuda model in Equations (1) and (2) is applied. Further, the pvT behavior of the testing materials is approximated by the Tait model in Equations (3)–(6). To finalize definition of the setup, the coefficients of the heuristic melt-conveying models are imported, including 51 and 69 constants in model 2D and model 3D, respectively.

**Figure 8:**
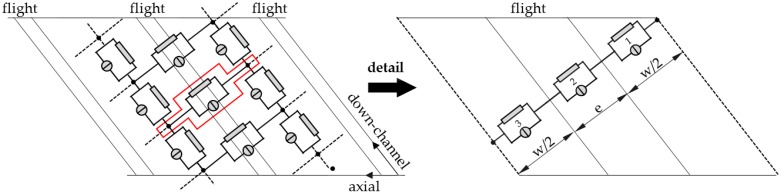
Network of a melt-conveying section. Cross-channel connections between nodal points were initialized with three elements connected in series and then replaced with one equivalent element.

In the second step, the geometry of the melt-conveying section is subdivided into a network of smaller segments of constant geometry. These sections are indicated by network elements (shown schematically in Figure 8, each of which consists of a source and a resistance that represent the local drag and pressure flows. The mass flow rate of each element is thus calculated by:

(14) $$\dot{m}={\dot{m}}_{d}+{\dot{m}}_{p}={\dot{m}}_{d}+k\Delta p$$

where the pressure flow is given by the conductance k and the pressure difference Δ*p*. Analogously to electrical circuits, the elements are connected by nodal points, whose axial and down-channel positions along the screw are functions of the outer screw diameter, the pitch angle and the number of elements per revolution. The last of these can be adjusted and defines the resolution of the grid. At each nodal point, the geometry of the screw channel is calculated. Note that this study includes the cross-channel flow over the screw flights, as captured by the network elements positioned perpendicular to the screw channel. To accurately describe the channel geometry in this direction, each cross-channel connection is initialized with three elements (with *h*_1_ = *h* and *w*_1_ = *w*/2, *h*_2_ = *δ* and *w*_2_ = *e*, *h*_3_ = *h* and *w*_3_ = *w*/2), which are then replaced by one equivalent element. The total conductance and drag flow for three elements connected in series is given by:

(15) $$\frac{1}{k_{\mathrm{total}}}=\sum_{i=1}^{3}\frac{1}{k_{i}}\;{\dot{m}}_{d,\mathrm{total}}=\left(\sum_{i=1}^{3}\frac{{\dot{m}}_{d,i}}{k_{i}}\right)\cdot k_{\mathrm{total}}$$

**Figure 9:**
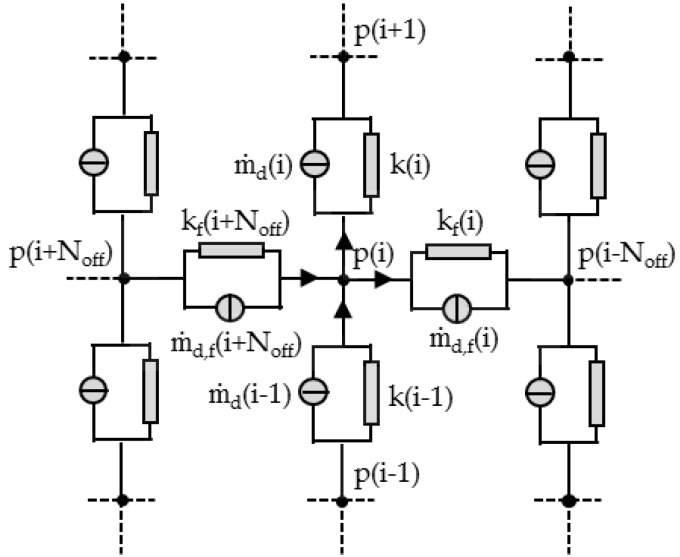
Equivalent circuit diagram.

To identify the pairs of nodal points connected in the cross-channel direction, a characteristic parameter was introduced based on geometric considerations. This parameter, which is referred to as *N*_off_, counts the number of down-channel elements between two nodal points that are connected in the cross-channel direction. The equivalent circuit diagram of the flow network is shown in Figure 9. At the beginning of the calculation, the pressures at the nodal points and the properties and flow rates of the network elements are initialized with zeroes.

In the third step, the equivalent circuit diagram is solved iteratively by means of nodal analysis. Assuming that the mass flow rates incident at a nodal point add up to zero (cf. Kirchhoff’s current law), the network equations are built at each node, as demonstrated by the following relationship for an arbitrary node with index *i*:

(16) $$\begin{array}{c} m_{d}\left(i-1\right)+k\left(i-1\right)\left(p\left(i-1\right)-p\left(i\right)\right)+{\dot{m}}_{d,f}\left(i+N_{\mathrm{off}}\right)+k_{f}\left(i+N_{\mathrm{off}}\right)\left(p\left(i+N_{\mathrm{off}}\right)-p\left(i\right)\right)\\ -{\dot{m}}_{d}\left(i\right)-k\left(i\right)\left(p\left(i\right)-p\left(i+1\right)\right)-{\dot{m}}_{d,f}\left(i\right)-k_{f}\left(i\right)\left(p\left(i\right)-p\left(i-N_{\mathrm{off}}\right)\right)=0 \end{array}$$

Taking all nodal points into account, the network equations are summarized in matrix form:

(17) $${\dot{m}}_{0}+K\cdot p=\dot{m}$$

where ${\dot{m}}_{0}$ is the drag flow vector, ***K*** the conductance matrix, ***p*** the pressure vector, and $\dot{m}$ includes the boundary conditions. For each simulation, we predefined the mass flow rate and calculated the pressure vector. The system is thus rearranged into:

(18) $$p=K^{-1}\left(\dot{m}-{\dot{m}}_{0}\right)$$

To solve the linear set of equations, the drag flow and conductance of each network element are determined as follows. First, the element dimensions are defined by using the geometric parameters known at each nodal point. The local flow properties are then evaluated. To this end, the Carreau-Yasuda data are converted into equivalent power-law parameters. On a log-log scale, the power law is a linear function and can be considered as the tangent of the Carreau-Yasuda model at a specific shear rate [35]. It is thus possible to determine the local power-law parameters from the Carreau-Yasuda parameters as follows:

(19) $$n_{\mathrm{pl}}=\frac{\left(\eta_{0}-\eta_{\infty }\right)\left(n_{\mathrm{cy}}-1\right){\left(a_{t}\lambda {\dot{\gamma }}_{\mathrm{rep}}\right)}^{a}{\left(1+{\left(a_{t}\lambda {\dot{\gamma }}_{\mathrm{rep}}\right)}^{a}\right)}^{\frac{n_{\mathrm{cy}}-1-a}{a}}}{\eta_{\infty }+\left(\eta_{0}-\eta_{\infty }\right){\left(1+{\left(a_{t}\lambda {\dot{\gamma }}_{\mathrm{rep}}\right)}^{a}\right)}^{\frac{n_{\mathrm{cy}}-1-a}{a}}}$$

(20) $$K=\eta_{\infty }+\left(\eta_{0}-\eta_{\infty }\right){\left(1+{\left(a_{t}\lambda {\dot{\gamma }}_{i}\right)}^{a}\right)}^{\frac{n_{\mathrm{cy}}-1}{a}}{\dot{\gamma }}_{\mathrm{rep}}^{1-n_{\mathrm{pl}}}$$

where the local effective shear rate is given by:

(21) $${\dot{\gamma }}_{\mathrm{rep}}=\sqrt{{\dot{\gamma }}_{x}^{2}+{\dot{\gamma }}_{z}^{2}}$$

and the shear-rate components in the cross- and down-channel directions are specified by a linear combination of a drag flow and a pressure flow.

**Figure 10:**
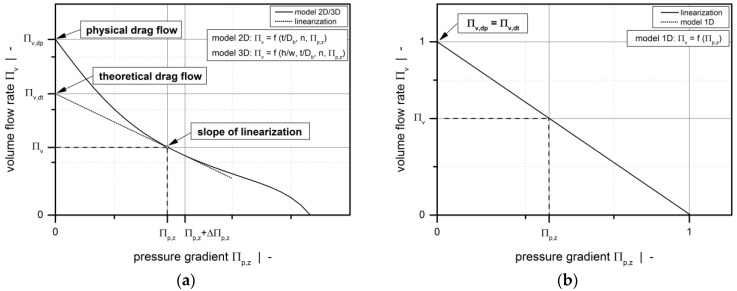
Linearization to the screw characteristic curve at operating point (*Π*_p,z_|*Π*_v_): Model 2D and 3D (**a**) and model 1D (**b**).

Finally, with the melt densities in the discretized screw channel being defined by the Tait equation, the local operating point (*Π*_p,z_, *Π*_v_) is evaluated using our melt-conveying models. For models 2D and 3D, a linearization of the screw characteristic curve is derived at this point, as shown in Figure 10a. For each element, theoretical drag flow and conductance are obtained from the initial value and the slope of the linearization, respectively. Considering the restrictive influence of the screw flights in model 2D requires these element properties to be corrected. To this end, the local shape factors obtained from Equations (12) and (13) are used.

Note that in the first iteration the flow in the metering section is governed solely by the drag flow components, as all pressure differences are set to zero. In each iterative step, the element properties and the nodal pressures are updated. A simulation was considered converged if the pressure difference between the first and the final nodal point was smaller than 0.01 bar. For all simulations, we used 200 elements per revolution to discretize the screw channel.

The section above explains the theoretical background of our modeling approach, combining the heuristic melt-conveying models and network theory. Due to the complexity of the analysis, however, presenting an example case of how the theory is applied for a particular set of input parameters goes beyond the scope of this contribution. The conductance matrix, for example, has more than 1,000,000 entries in the analysis presented, which is moreover updated each iteration.

### 3.3. Modified Newtonian Pumping Model

In addition to comparing the heuristic melt-conveying models to experimental data, we tested the theories against a Newtonian pumping model well known from the literature [2,6]. This classical approach, which is referred to as model 1D, describes the flow in a one-dimensional metering channel as a linear superposition of a drag and a pressure flow. In the following analysis, the approach is used as a reference model to be able to assess the advantages and disadvantages of the new melt-flow theories. Using dimensionless notation, the output-pressure gradient relationship is written as:

(22) $$\mathrm{model}\;1D:\;\Pi_{v}=1-\Pi_{p,z}$$

where *Π*_v_ is defined in Equation (10). In the Newtonian case, the dimensionless pressure gradient is reduced to:

(23) $$\Pi_{p,z}=\frac{h^{2}p_{z}^{\prime }}{6\eta_{\mathrm{rep}}v_{b,z}}$$

Traditionally, the model shown above is based on constant viscosity. In the discretized screw channel, however, the Newtonian viscosity is updated locally for each network element by using the corresponding local shear rate and the Carreau-Yasuda model. Further, since the screw characteristic curve is a linear function, the element properties (drag flow and conductance) are derived directly from the model itself, as indicated in Figure 10b. Similarly, to include the influence of the screw flights on the pumping characteristics, the drag flow and conductance are reduced by applying the local shape factors for each element.

## 4. Results and Discussion

Employing our screw-simulation routine, we replicated the extruder tests carried out in the first part of this study. To this end, we predefined the screw speed and the measured extrusion data (mass flow rate and melt temperature) and predicted the axial pressure profiles along the metering zone, which starts at an axial position of approximately 17.2·*D*_b_. For convenience, the pressure profiles were shifted to the level of the discharge pressure measured at the screw tip.

**Figure 11:**
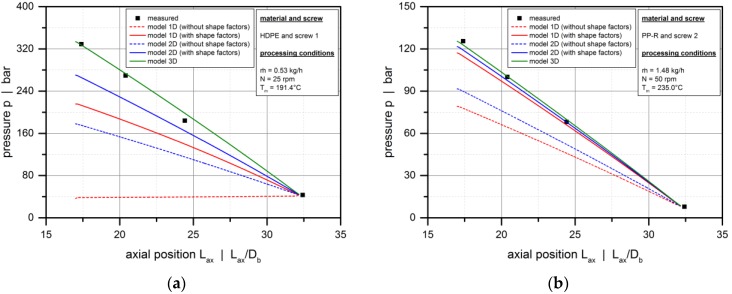
Influence of the screw flights. Comparison between experimental data and solutions from model 1D, model 2D, and model 3D. Axial pressure profiles for various experimental setups: HDPE, screw 1, *N* = 25 rpm (**a**); PP-R, screw 2, *N* = 50 rpm (**b**).

Figure 11 shows the influence of the flight flanks on the pressure characteristics of the metering zone by comparing the experimental results with the solutions obtained from the heuristic models (model 2D and 3D) and the modified Newtonian model (model 1D). With the effect of the screw flights being automatically included in model 3D, model 1D and 2D were used with and without shape factors. For all experimental setups, the melt-conveying zone is overridden, as indicated by a negative pressure gradient. This means that the pressure generated in the compression section decreases along the metering zone. Clearly, with the bypass valve fully open, the extruder was operated at open discharge, causing the pressure to reduce towards the extruder end.

The pressure profiles obtained from model 3D are in excellent agreement with the measured data. Considering the three-dimensional screw channel and the shear-thinning flow behavior of the polymer melt, this model accurately represents—both qualitatively and quantitatively—the pressure-consuming conveying behavior under all processing conditions. In contrast, applying model 2D without shape factors underestimates the experimental pressure drop significantly. Obviously, the two-dimensional theory describes the flow in infinitely wide screw channels. The rate-reducing influence of the screw flights is thus ignored, which causes the drag flow to be overestimated. As a result, a lower negative pressure gradient is predicted to achieve the same throughput.

The least accurate results are obtained if model 1D is used without shape factors. For this Newtonian approach, the dimensionless drag flow is fixed at *Π*_v,d_ = 1.0, as shown in Figure 10b. Hence, neither the influence of the screw flights nor the effect of the cross-channel flow is taken into account. For shear-thinning polymer melts, the cross-channel flow has a major impact on the conveying characteristics: Increasing the pitch-to-diameter ratio of the screw reduces the drag flow in the screw channel. Consequently, model 1D overestimates the drag flow even more significantly than model 2D, and we observe a pressure-generating conveying behavior rather than an overridden melt-conveying section for the operating conditions illustrated in Figure 11a. Clearly, the accuracy of the modified Newtonian model increases with decreasing shear-thinning of the polymer melt, as shown in Figure 11b.

**Figure 12:**
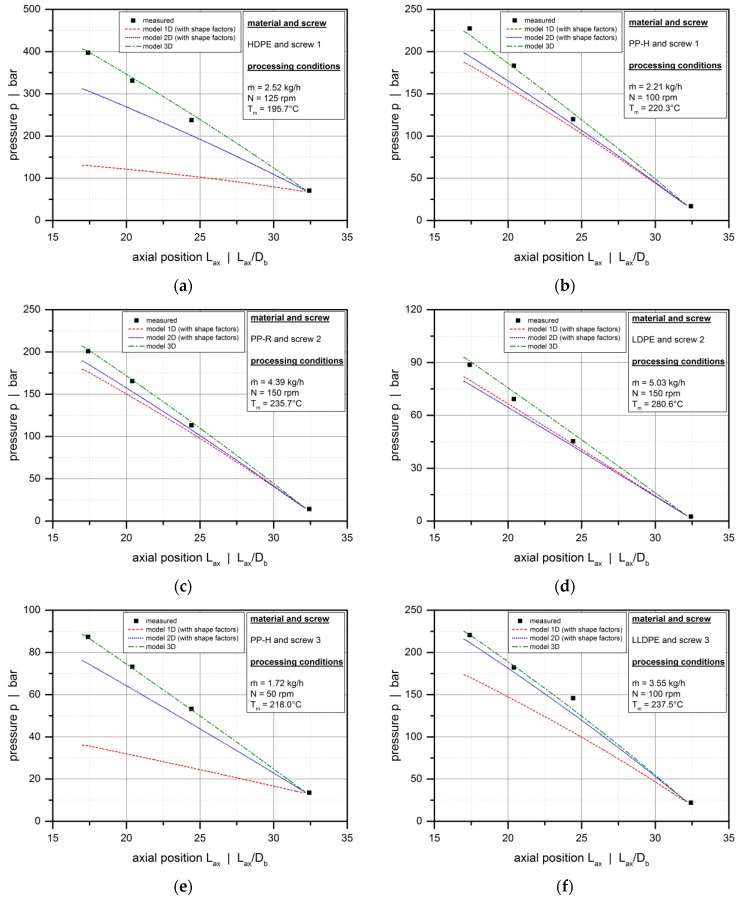
Comparison between experimental data and solutions from model 1D, model 2D, and model 3D. Axial pressure profiles for various experimental setups: HDPE, screw 1, *N* = 125 rpm (**a**); PP-H, screw 1, *N* = 100 rpm (**b**); PP-R, screw 2, *N* = 150 rpm (**c**); LDPE, screw 2, *N* = 150 rpm (**d**); PP-H, screw 3, *N* = 50 rpm (**e**); LLDPE, screw 3, *N* = 100 rpm (**f**).

Including the shape factors in model 1D and 2D significantly increases the accuracy of the analyses, as shown in Figure 12 for various extrusion conditions. The most accurate predictions, however, are obtained from model 3D for all setups.

The deviations between model 3D and model 2D (with shape factors) can be explained by the nature of the correction parameters, which originate from the classical Newtonian pumping model [2]. For shear-thinning polymer melts, the influence of the screw flights is more pronounced than for Newtonian fluids. This effect is accurately captured in model 3D. In contrast, applying the Newtonian shape factors to model 2D underestimates the effect of the screw flights on flow.

**Figure 13:**
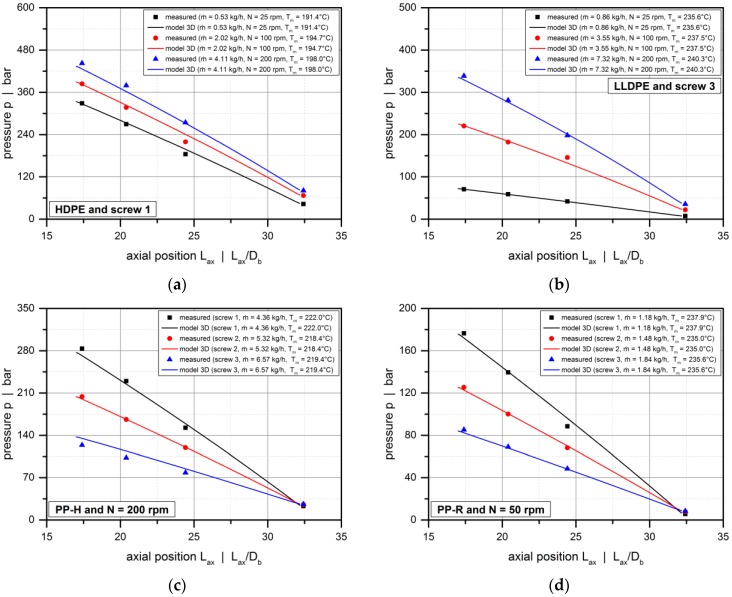

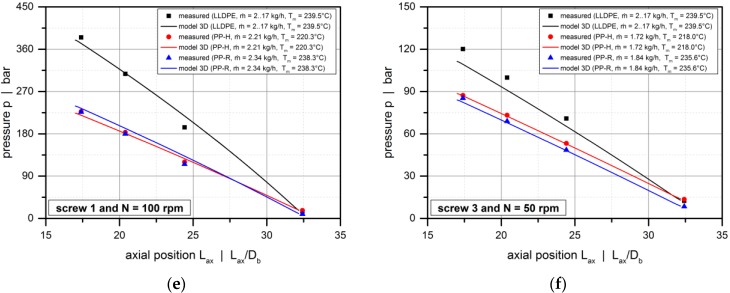
Comparison between experimental data and solutions resulting from model 3D. Axial pressure profiles for various experimental setups: HDPE, screw 1 (**a**); LLDPE, screw 3 (**b**); PP-H, *N* = 200 rpm (**c**); PP-R, *N* = 50 rpm (**d**); screw 1, *N* = 100 rpm (**e**); screw 3, *N* = 50 rpm (**f**).

To further investigate the validity of model 3D, we compared experimental and calculated pressure profiles for additional setups (Figure 13), analyzing the influences of screw speed (a,b), screw design (c,d), and material (e,f). For all setups, the pressure drops predicted by model 3D match the experimental data. Although we observe minor local deviations which may result from measurement errors, the general pressure characteristics are reflected accurately. Given the variety of screw designs, materials, and processing conditions used in this study, the validity of model 3D is confirmed for a wide application range. Note that, due to the dimensionless form of the melt-flow theories, the results are not restricted to the size of the laboratory extruder, but can be scaled up to larger machines. In general, the findings are valid for all dimensional variations that result in the given sets of dimensionless input parameters.

Employing the three-dimensional melt-conveying model in combination with network theory enables fast and stable prediction of the conveying characteristics of polymer melts in metering channels. Owing to the algebraic structure of the output-pressure gradient relationship used in the network model, the approach can be easily implemented in practice, removing the need for time-consuming and computationally expensive numerical procedures such as the finite-element method (FEM) or finite-volume method (FVM). Although the research approach is not capable of outperforming these numerical methods in terms of accuracy, as it rather approximates the numerical solutions for a large set of design points, it is considerably faster than solving the flow equations numerically. The modeling approach thus provides a useful tool for quickly analyzing the pumping behavior of various screw designs, which is especially important in optimization studies.

For further model optimization, we are currently working on a modification that includes the curvature of the screw. For large height-to-diameter ratios (e.g., in the melt channel of barrier screws), the curvature of the screw must be considered, as the flat-plate approximation underestimates the flow rate under most processing conditions. Over-estimation is observed only for high back pressures [31]. Additionally, the models are being implemented in a non-isothermal screw-calculation routine considering a temperature-dependent viscosity. In the extrusion of high-molecular-weight polymers, viscous dissipation plays an important role because, due to inner friction, mechanical energy is transformed into heat. Especially at high screw speeds, this effect leads to a pronounced axial melt temperature increase that locally decreases viscosity, thus affecting the pumping characteristics of the screw.

## 5. Conclusions

This study has investigated the validity of our recently proposed two- and three-dimensional heuristic melt-flow theories by comparing the models to experimental extrusion data. In the first part, extruder tests were performed on a 19/33D laboratory single-screw extruder. Employing various standard extruder screws, materials, and processing conditions, we analyzed a great number of extrusion conditions, for which we measured mass flow rates, axial pressure profiles, and melt temperatures. In the second part, a network-theory-based flow-simulation routine using our heuristic melt-conveying models was developed in MATLAB to reproduce the conveying behavior of the metering zones in silico. Furthermore, a modified Newtonian pumping model was implemented as a reference model. Leakage flow over the screw flights was considered in the discretization of the screw channel.

Applying our screw-simulation routine, we calculated the axial pressure profiles for all experimental setups. The results of the three-dimensional heuristic approach are in excellent agreement with the measured values, which confirms the validity of the model for a large range of applications. The solutions of the two-dimensional heuristic approach, in contrast, deviate from the experimental data, as the model ignores the rate-limiting influence of the screw flights. When including shape factors, however, the accuracy can be significantly increased. The modified Newtonian pumping model yields the least accurate results.

We conclude that the two-dimensional theory without shape factors remains highly relevant in the case of high-performance screws with an undercut in the flight. In such cases, the influence of the flight is significantly smaller than in standard extruder screws. This topic is currently being investigated in a follow-up project. Depending on the flight geometry, we expect to propose a combination of models 2D and 3D.

## Figures and Tables

**Table 1:** Comparison of approximate melt-conveying models for power-law fluids.

Year	Melt-Conveying Model	Flow Situation	Transverse Flow	Flight Flanks	Range of Pressure Gradients
1981	Booy [16]	2D	✓	-	positive
2001	White and Potente [3]	2D	✓	-	negative and positive
2014	Rauwendaal [6]	2D	✓	-	positive
2017	Pachner et al. [20]	2D	✓	-	negative and positive
2017	Marschik et al. [21]	3D	✓	✓	negative and positive

**Table 2:** Comparison of the materials tested (MFR—melt flow rate, HDPE—high density polyethylene, LLDPE—linear low density polyethylene, PP-H—polypropylene homopolymer, PP-R—polypropylene random copolymer, LDPE—low density polyethylene).

Material	Type	MFR (ISO 1133)		Application	Manufacturer
HE3490-LS	HDPE	0.25 g/10 min	(190 °C, 5 kg)	pipes	Borealis
2107GC	LLDPE	2.30 g/10 min	(190 °C, 2.16 kg)	films	Dow
HC205TF	PP-H	4.00 g/10 min	(230 °C, 2.16 kg)	thermoforming	Borealis
RD204CF	PP-R	8.00 g/10 min	(230 °C, 2.16 kg)	films	Borealis
CA9150	LDPE	15.0 g/10 min	(190 °C, 2.16 kg)	coatings	Borealis

**Table 3:** Carreau-Yasuda and Tait model parameter values.

	Parameter	Unit	HDPE	LLDPE	PP-H	PP-R	LDPE
Carreau Yasuda	*η*_0_	Pas	320,046	3898	8234	3252	2474
	*η*_∞_	Pas	0	0	0	0	0
	*λ*	s	9.178	0.003	0.129	0.063	0.006
	*n*_cy_	-	0.196	0.000	0.217	0.263	0.004
	*a*	-	0.570	0.385	0.468	0.595	0.248
	*α*	-	0.024	0.009	0.025	0.017	0.020
	*T*_0_	K	473.15	473.15	473.15	473.15	473.15
Tait	*b*_1m_	m^3^/kg	0.00127	0.00130	0.00128	0.00128	0.00132
	*b*_2m_	m/(kg·K)	9.03 × 10^−7^	9.50 × 10^−7^	8.42 × 10^−7^	1.01 × 10^−6^	9.95 × 10^−7^
	*b*_3m_	Pa	63,645,134	45,451,967	59884,263	63,374,356	93,840,512
	*b*_4m_	1/K	64 × 10^−5^	75 × 10^−5^	0	355 × 10^−5^	337 × 10^−5^
	*b*_5m_	K	393.96	383.13	405.83	395.10	370.15
	*b*_6m_	K/Pa	2.99 × 10^−7^	2.50 × 10^−7^	3.05 × 10^−7^	2.50 × 10^−7^	2.34 × 10^−7^

**Table 4:** Barrel temperature profiles in °C.

Material	*T*_1_	*T*_2_	*T*_3_	*T*_4_
HDPE	200	200	200	200
LLDPE	250	250	250	250
PP-H	230	230	230	230
PP-R	250	250	250	250
LDPE	300	300	300	300

**Table 5:** Dimensions of extruder screws.

Dimensions		Unit	Screw 1	Screw 2	Screw 3
outer diameter	*D*_s_	mm	19	19	19
pitch	*t*	mm	15	19	25
pitch angle	*φ*_b_	°	14.11	17.66	22.73
flight width	*e*	mm	3	3	3
channel width	*w*	mm	11.55	15.10	20.06
channel depth (feeding zone)	*h*_f_	mm	3.9	3.9	3.9
channel depth (metering zone)	*h*	mm	1.4	1.4	1.4
compression ratio	*κ*	-	2.79	2.79	2.79
flight clearance	*δ*	mm	0.05	0.05	0.05
number of parallel flights	*i*	-	1	1	1
pitch-to-diameter ratio	*t*/*D_s_*	-	0.79	1.00	1.32
aspect ratio (metering zone)	*h*/*w*	-	0.12	0.09	0.07

**Table 6:** Comparison of the heuristic melt-conveying models.

Modeling Steps		Model 2D	Model 3D
**step 1**	**geometry**		
	▪ representation	flat-plate model with moving barrel	
	▪ coordinate system	Cartesian	
	▪ leakage flow	no	
	▪ effect of flight flanks	no	yes
	**fluid**		
	▪ viscosity function	power-law fluid	
	▪ density behavior	incompressible	
	**flow situation**		
	▪ time-dependency	stationary	
	▪ thermal effects	isothermal	
	▪ inlet/outlet effects	fully developed	
	▪ wall adhesion	yes	
	▪ gravitational forces	no	
	▪ Reynolds number	*Re* << 1	
	▪ velocity field	two-dimensional	three-dimensional
	**governing equations**		
	▪ constitutive equation	*τ* = 2·*η* **D** with **D** = 1/2·(∇**v** + ∇**v**^T^)	
	▪ continuity equation	fulfilled	*∂v*_x_/*∂x* + *∂v*_y_/*∂y* = 0
	▪ x-momentum equation	*∂p*/*∂x* = *∂τ*_yx_/*∂y*	*∂p*/*∂x* = *∂τ*_xx_/*∂x* + *∂τ*_yx_/*∂y*
	▪ y-momentum equation	-	*∂p*/*∂y* = *∂τ*_xy_/*∂x* + *∂τ*_yy_/*∂y*
	▪ z-momentum equation	*∂p*/*∂z* = *∂τ*_yz_/*∂y*	*∂p*/*∂z* = *∂τ*_xz_/*∂x* + *∂τ*_yz_/*∂y*
**step 2**	**theory of similarity**		
	▪ output parameter	*Π*_v_	
	▪ input parameters	*t*/*D*_b_, *n*, *Π*_p,z_	*t*/*D*_b_, *n*, *Π*_p,z_, *h*/*w*
	**numerical solution**		
	▪ method	Newton-Raphson	finite-volume method
	▪ number of design points	10,000	87,840
**step 3**	**analytical approximation**		
	▪ method	symbolic regression based on genetic programming	
	▪ approximation	*Π*_v_ = *f*(*t*/*D*_b_, *n*, *Π*_p,z_)	*Π*_v_ = *f*(*t*/*D*_b_, *n*, *Π*_p,z_, *h*/*w*)
	▪ function set	+, −, ·, /, sin, cos, exp, log	+, −, ·, /, sin, cos
	▪ constants	51	69

## Appendix A

**Table A1:** Experimental data.

Operating Point	Screw	Material	Screw speed (rpm)	Throughput (kg/h)	Melt Temperature (°C)	Pressure Profiles (Figure Number)
1	1	HDPE	25	0.53	191.4	Figure 11a
2	1	HDPE	125	2.52	195.7	Figure 12a
3	1	PP-H	100	2.21	220.3	Figure 12b
4	1	HDPE	25	0.53	191.4	Figure 13a
5	1	HDPE	100	2.02	194.7	Figure 13a
6	1	HDPE	200	4.11	198.0	Figure 13a
7	1	PP-H	200	4.36	222.0	Figure 13c
8	1	PP-R	50	1.18	237.9	Figure 13d
9	1	LLDPE	100	2.17	239.5	Figure 13e
10	1	PP-H	100	2.21	220.3	Figure 13e
11	1	PP-R	100	2.34	238.3	Figure 13e
12	2	PP-R	50	1.48	235.0	Figure 11b
13	2	PP-R	150	4.39	235.7	Figure 12c
14	2	LDPE	150	5.03	280.6	Figure 12d
15	2	PP-H	200	5.32	218.4	Figure 13c
16	2	PP-R	50	1.48	235.0	Figure 13d
17	3	LLDPE	50	2.17	239.5	Figure 13f
18	3	PP-H	50	1.72	218.0	Figure 13f
19	3	PP-R	50	1.84	235.6	Figure 13f
20	3	PP-H	50	1.72	218.0	Figure 12e
21	3	LLDPE	100	3.55	237.5	Figure 12f
22	3	LLDPE	25	0.86	235.6	Figure 13b
23	3	LLDPE	100	3.55	237.5	Figure 13b
24	3	LLDPE	200	7.32	240.3	Figure 13b
25	3	PP-H	200	6.57	219.4	Figure 13c
26	3	PP-R	50	1.84	235.6	Figure 13d
